# Quality of intrapartum care by skilled birth attendants in a refugee clinic on the Thai-Myanmar border: a survey using WHO Safe Motherhood Needs Assessment

**DOI:** 10.1186/s12884-015-0444-0

**Published:** 2015-02-05

**Authors:** Gabie Hoogenboom, May Myo Thwin, Kris Velink, Marijke Baaijens, Prakaykaew Charrunwatthana, François Nosten, Rose McGready

**Affiliations:** Shoklo Malaria Research Unit, Mahidol-Oxford Tropical Medicine Research Unit, Faculty of Tropical Medicine, Mahidol University, Mae Sot, Thailand; AVAG Midwifery Academy Amsterdam Groningen, Amsterdam, The Netherlands; Mahidol-Oxford Tropical Medicine Research Unit (MORU), Faculty of Tropical Medicine, Mahidol University, Bangkok, Thailand; Centre for Tropical Medicine and Global Health, Nuffield Department of Medicine, University of Oxford, Oxford, UK

**Keywords:** Skilled birth attendant, Health care quality assessment, Pregnancy and childbirth care, Safe motherhood, Refugee setting

## Abstract

**Background:**

Increasing the number of women birthing with skilled birth attendants (SBAs) as one of the strategies to reduce maternal mortality and morbidity must be partnered with a minimum standard of care. This manuscript describes the quality of intrapartum care provided by SBAs in Mae La camp, a low resource, protracted refugee context on the Thai-Myanmar border.

**Methods:**

In the obstetric department of Shoklo Malaria Research Unit (SMRU) the standardized WHO Safe Motherhood Needs Assessment tool was adapted to the setting and used: to assess the facility; interview SBAs; collect data from maternal records during a one year period (August 2007 – 2008); and observe practice during labour and childbirth.

**Results:**

The facility assessment recorded no ‘out of stock’ or ‘out of date’ drugs and supplies, equipment was in operating order and necessary infrastructure e.g. a stand-by emergency car, was present. Syphilis testing was not available. SBA interviews established that danger signs and symptoms were recognized except for sepsis and endometritis. All SBAs acknowledged receiving theoretical and ‘hands-on’ training and regularly attended deliveries. Scores for the essential elements of antenatal care from maternal records were high (>90%) e.g. providing supplements, recording risk factors as well as regular and correct partogram use. Observed good clinical practice included: presence of a support person; active management of third stage; post-partum monitoring; and immediate and correct neonatal care. Observed incorrect practice included: improper controlled cord traction; inadequate hand washing; an episiotomy rate in nulliparous women 49% (34/70) and low rates 30% (6/20) of newborn monitoring in the first hours following birth. Overall observed complications during labour and birth were low with post-partum haemorrhage being the most common in which case the SBAs followed the protocol but were slow to recognize severity and take action.

**Conclusions:**

In the clinic of SMRU in Mae La refugee camp, SBAs were able to comply with evidence-based guidelines but support to improve quality of care in specific areas is required. The structure of the WHO Safe Motherhood Needs Assessment allowed significant insights into the quality of intrapartum care particularly through direct observation, identifying a clear pathway for quality improvement.

**Electronic supplementary material:**

The online version of this article (doi:10.1186/s12884-015-0444-0) contains supplementary material, which is available to authorized users.

## Background

In an attempt to reduce maternal mortality and morbidity, global efforts have been directed towards increasing access to quality maternal health care services by increasing the number and competence of skilled birth attendants (SBAs) [1-3]. The World Health Organization (WHO) defines an SBA as “an accredited health professional such as midwife, doctor or nurse who has been educated and trained to proficiency in the skills needed to manage normal (uncomplicated) pregnancies, childbirth and the immediate postnatal period, and in the identification, management and referral of complications in women and newborns” [4]. In reality, SBAs vary widely in terms of the type of health care providers, job description and responsibilities [5]. Women’s perception of the competency of SBAs and quality of the health care facility influences access [6]. Hence, training of SBAs using evidence-based guidelines such as the WHO Integrated Management of Pregnancy and Childbirth (IMPAC) [7] including emergency obstetric and neonatal care (EmONC) is vital [8]. Maternal and neonatal mortality and morbidity statistics poorly reflect the quality of maternal health care services [9] and assessment of the process of care is important [10].

The first wave of refugees on the Thai-Myanmar border started arriving in 1986 and now refugee camps dot the border over an expanse of more than 500 km. In the camps’ early years traditional birth attendants were the primary providers of obstetric care. As the humanitarian emergency evolved to a more protracted refugee situation, a greater proportion of care has been provided by SBAs [11]. The availability and quality of care in the vicinity was previously assessed during a 5 year (2000–2005) project aimed at increasing availability of EmONC in conflict-affected settings [12]. In the three refugee camps involved in the project: Umpiem Mai, Nu Po and Ban Don Yang, women requiring any EmONC were referred out of the camp to the nearest Thai Hospital. In Mae Tao Clinic which provides health care services for migrants and internally displaced populations the availability and quality of care were described as inadequate with shortages of infrastructure, equipment, staff, medicines and training at the start of the program with later improvement.

The focus of this paper is on an initial assessment of the quality of intrapartum care provided by SBAs in Mae La refugee camp (estimated population of 46,000), the largest of the refugee camps on the Thai-Myanmar border [13]. A standardized tool is used, the WHO Safe Motherhood Needs Assessment (SMNA) [14], which uses the WHO Mother-Baby package as a minimal set of interventions [15]. The objective is to describe the quality of process of care, including aspects as infrastructure, equipment, training and supervision, and including real time observation of SBA performances during the intrapartum period. The subsequent steps of the clinical audit cycle will be published separately.

## Methods

### Population and background

Mae La refugee camp has had at least four different non-governmental organizations (NGOs) providing health care over a 28 year period. The education level in the Mae La camp population is low, with 37% having no formal education at all and only 6% having completed standard 10 (secondary education) [16]. Training of health workers in this setting has been ad hoc, often responding to immediate needs. Currently Première Urgence–Aide Médicale International (PU-AMI) is the main health care provider while Shoklo Malaria Research Unit (SMRU) has been providing antenatal and childbirth services since 1995. Antenatal care (ANC) coverage is high with more than 90% of pregnant women living in Mae La camp attending at least once [17], while more than 75% of women in Mae La camp give birth in the SMRU birth centre with SBAs [18].

SBAs in the SMRU clinic are literate residents from the Mae La camp population. Their background may include health training from other NGO educators in the border region, or from Myanmar. Some have completed high school without having other health training. SMRU provides additional internal training for all SBAs. The curriculum for SMRU SBAs was formalized in 2010. The birth centre is open 24 hours per day with week-day attendance by an expatriate medical doctor with obstetric experience who is available after-hours for consultations by telephone. Evidence-based guidelines have been available since 1999 and eight out of the nine WHO EmONC signal functions are available on site (with the exception of caesarean section) [19]. Challenges being faced are high staff turn-over due to resettlement and return, low theoretical background knowledge, and daily work that requires a high level of competence. The quality of care provided by SBAs was identified as a problem and a clinical audit cycle commenced [20].

### Components of the Safe Motherhood Needs Assessment (SMNA)

**Figure 1:**
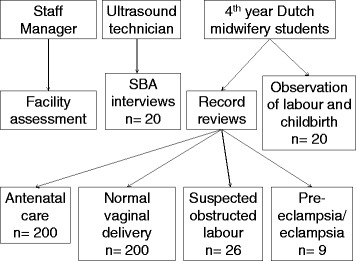
**Overview of the components of SMNA and who carried them out.**

Following the SMNA guideline a core team, in this case two obstetric doctors and two staff managers, were appointed to conduct a policy and management assessment. Using a list of guiding questions from the SMNA guideline the core team analysed the local structure of health care services. This information was used to make a selection from the provided survey forms, which were then adapted to the local situation of the SMRU clinic in Mae La refugee camp. The selected survey forms included four components: the facility assessment, midwife interviews (adapted to SBA interviews), record reviews and observations of deliveries (Figure 1). Antenatal and post-partum client interviews were left out as the primary interest was not patient satisfaction. The traditional birth attendant (TBA) interview was also excluded as TBAs are not trained by SMRU although the pregnant women were welcome to invite anyone, including TBA, as their support person into the birth room.

The questions on the selected forms were examined by the core team for usefulness and appropriateness. Questions with direct operational relevance for the setting were added. These adapted survey forms were piloted at another SMRU clinic for Karen and Burmese migrants (30 km south of Mae La) to ensure there were no unanticipated difficulties (Additional file 1). A surveyor manual was developed and training sessions for the data collection were provided as described in the SMNA guidelines. As far as we are aware there have been no publications on the reliability and validation of the SMNA.

#### Facility assessment

The facility assessment was done by a staff manager employed by SMRU and fluent in Burmese and English. The staff manager was chosen because of her ability to comprehend the scope of the task and her independence from the birth centre staff, as she was not a clinic employee. The facility assessment included an interview with the SBA in charge of the birth centre and observations of equipment, supplies and medicines at the clinic.

#### SBA interviews

SBA interviews were done by an SMRU ultrasound technician chosen for practical reasons including: fluency in Karen, Burmese and English; ability to comprehend the scope of the task; independent from the birth centre staff; and friendly demeanour.

A group explanation to SBAs about the research project was done with information about the interviews and observations. It was emphasized that both their answers and their verbal consent or refusal would be anonymous. The ultrasound technician asked the SBAs individually for verbal consent and if consent was provided, then conducted the interview. All 20 SBAs working in the birth centre at the time agreed to participate.

#### Record reviews and observation of labour and child birth

Two 4th year female Dutch midwife students conducted the record reviews. They were chosen because they could follow the detailed instructions required for data extraction. The sampling period for record review was from August 1st 2007 until July 31st 2008. Following the SMNA guidelines we sampled randomly 200 ANC records, 200 out of 1042 (19%) normal vaginal delivery (NVD) records of women delivered at SMRU, records of all cases of pre-eclampsia and eclampsia and obstructed labour, nine and 26 cases respectively.

The same two 4th year Dutch midwife students conducted the observation of deliveries and were chosen because they knew correct midwifery practice and as they were not part of the usual birth team they were well placed to observe and not intervene. It was stressed to the SBA that being observed was voluntary and that they could exchange with a SBA working in the ward if they didn’t want to be observed in the birthing room. Prior to any observations of labour and childbirth verbal consent was obtained from each pregnant woman by the SBA on behalf of the Dutch midwife student. All participants were assured they had the right to withdraw their consent at any time. It was explained that they would receive the same care as all other patients regardless of their participation or refusal. As around 50% of the mothers are illiterate [18] the information and consent were done verbally for all women. All 20 women that gave birth while the observers were present during the study period were asked to participate and they all agreed to be observed during birthing.

As the SNMA does not provide a survey form for the observation of deliveries, a checklist was developed that included aspects of hygiene, communication, monitoring, midwifery skills and EmONC skills [21]. These checklists were used as a guide to observe the skills of the SBA team that conducted the birth and examples of situations and information from the birth record were used to complete the data. The data were collected over a period of six weeks in November – December 2008.

### Ethical approval

This observational study was undertaken from September 2008 until February 2009. Ethical approval for the SMNA was granted by the Research Ethics Committee of the Royal Tropical Institute in Amsterdam, The Netherlands and by the local Tak Province Border Community Ethics Advisory Board and for retrospective analysis of pregnancy records from the Oxford Tropical Research Ethics Committee (OXTREC 28–09).

### Statistical analysis

All quantitative data were entered, validated, analysed and interpreted using Epi-Info Version 6 statistical software following SMNA guidelines. Data were tabulated and described in terms of proportions when appropriate. Record reviews and observation of labour and childbirth components of the SMNA were reported together as they both overlapped and complemented each other.

## Results

### Facility assessment

The facility assessment showed that all essential equipment for standard ANC, basic EmONC and essential care of obstetric complications were present at SMRU clinic in Mae La, except for syphilis testing kits. All supplies were available from a routine procurement system. For comprehensive EmONC the essential materials for the provision of donor blood for transfusion were in place, but storage facilities for blood bank services were not available. A referral car for obstetric emergencies was present 24 hours per day.

The essential elements of ANC (except syphilis testing) were all performed with high accuracy (Table 1).

**Table 1:** **Results ANC record review**

**Recorded on the ANC card**	**Frequency (n = 200)**	**Percentage %**
Gravida	189	94.5
Results of at least one urine test	192	96
Results of haematocrit test	199	99.5
Results of syphilis test	0	0
Supplementation with iron/folic acid	200	100
Results of malaria test	200	100
Provision of malaria treatment (if test positive)	10	100
Risk factors	200	100
HIV test (provider initiated)	195	97.5
HIV post-test counselling	188	94.0

### SBA interviews

The 20 SBAs working in Mae La were all asked to identify spontaneously which danger signs and symptoms in patients would prompt them to inform the doctor (Table 2). In general they recognised danger signs well, except for sepsis and endometritis. All SBAs acknowledged receiving theoretical and ‘hands-on’ training, including personal supervision. Family planning training had been provided six months earlier for 11 of the SBAs and the other nine had never received any training on this subject. All SBAs attended deliveries regularly and when asked directly all but two (10%) had attended their last birth within one month and 12 (60%) of them within the past week. Each SBA reported attending each of these obstetric complications that required life saving skills (post-partum haemorrhage (PPH), obstructed labour, puerperal sepsis, (pre-) eclampsia and abortion complications) at least once in the previous six months. Only six SBAs had not managed to care for a (pre-) eclampsia case in the six months preceding the interview. Two thirds (12) of all SBAs reported that they did not feel confident when doing their work.

**Table 2:** **Recognition of danger signs by SBAs**

**Danger sign**	**SBAs that would discuss this with the doctor % (n)**
Previous bad obstetric history/caesarean section/stillbirth	85 (17)
Hypertension/headache /oedema/seizures	95 (19)
Anaemia/pallor/fatigue/dyspnoea	70 (14)
Fetal distress/no fetal movement	100 (20)
Abnormal lie/position of fetus	90 (18)
Sepsis/smelly discharge/post-partum abdominal pain	30 (6)
Slight bleeding/spotting	65 (13)
Haemorrhage/heavy bleeding	85 (17)
Twins/large abdomen	70 (14)
Obstructed/prolonged labour/indication for vacuum	100 (20)
Asthma/fever/malaria/HIV positive	85 (17)
(Grand)multiparity/premature labour/abnormal baby	95 (19)

### Observations of labour and child birth and record reviews

The SBAs worked in teams of three SBAs with different levels of experience, working together to provide support for a woman during childbirth. In general the most senior SBA was the team leader and conducted the actual birth, including active management of the third stage of labour (AMTSL) and suturing. Less experienced SBAs cut the cord and gave immediate neonatal care, monitored the woman’s vital signs, contractions and the fetal heart beat and wrote these on the partograph and prepared medication. More SBAs were involved when complications and emergencies occurred.

#### Monitoring during labour and childbirth

All 20 women observed during labour and childbirth were asked about their general health, obstetric history and their risk factors were identified. This was also the case in all reviewed records. The partograph was always used to monitor the progress of labour. Each woman had a vaginal examination on admission and at least 4 hourly afterwards and this was likewise confirmed in the record reviews. Monitoring of the fetal heart beat at least on an hourly basis was done in 100% (20/20) of the observed women and in 95% (190/200) of the record reviews. Monitoring of the blood pressure at least once per hour was done in 40% (8/20) of the observed labours and in 45% (89/200) of the record reviews (Table 3).

**Table 3:** **Results observations and NVD record reviews**

	**Observations (20)**		**Record reviews (200)**	
	**n**	**%**	**n**	**%**
**Monitoring during labour and childbirth**				
History and risk factors (ANC card)	20	100	200	100
Progress of labour (partograph)	20	100	200	100
Vaginal examinations at least 4 hourly	20	100	200	100
Monitoring of fetal heart beat at least 1 hourly	20	100	190	95
Monitoring of blood pressure at least 1 hourly	8	40	89	45
**Care during labour and childbirth**				
Support person present	20	100	N/A	N/A
Hand washing	15	75	N/A	N/A
Urine catheter	6	30	38	19
Correct use of AMTSL	14	70	N/A	N/A
Time of birth of the placenta	20	100	0	0
Examination of placenta for completeness	15	75	92	46
Episiotomy	4	20	39	20
Correct inspection of perineum and suturing	17	85	N/A	N/A
Measure and estimate blood loss	18	90	8	4
**Post-partum care**				
Mother stays 2 hours in birth room	2	10	N/A	N/A
Monitoring of vital signs and blood loss within 2 hours	19	95	N/A	N/A
**Neonatal care**				
Clean environment	20	100	N/A	N/A
Immediate care of the newborn within 1 hour	20	100	N/A	N/A
Assess apgar scores	20	100	200	100
Clean cord care	20	100	N/A	N/A
Measure birth weight	20	100	200	100
Monitoring of vital signs of baby	6	30	N/A	N/A
**Complications**				
Vacuum assisted birth	1	5	N/A	N/A
Shoulder dystocia	1	5	N/A	N/A
Pre-eclampsia	1	5	N/A	N/A
Post-partum haemorrhage (blood loss > 500ml)	4	20	11	6
Post-partum infection	3	15	10	5
Referral to higher level of care	1	5	N/A	N/A

N/A = not applicable, as this cannot be extracted from a patient record.

#### Support and hygiene

One support person invited by the pregnant woman was encouraged to stay with the woman during childbirth while most of the active phase of labour was conducted outside the birth room and more than one person could attend the woman at this stage. In 11 out of 20 births the woman was accompanied by a TBA into the birth room, seven women invited a female relative and in two deliveries the husband provided support. The support person often did not stay for the entire third stage of labour, but left as soon as the baby was ready to be shown to the family waiting outside the birth room. During the birth the SBAs could forget to notify and explain to the woman the necessary medical interventions especially when busy. During complications and emergencies informing and reassuring the mother was minimal.

In 15 of the births at least one SBA washed her hands before and/or after the birth, but in only one of the 20 observed births all SBAs washed their hands, and this occurred after, not before the birth. Overall attention to hand washing was poor, although water, soap and sinks were available in the birth room.

A urine catheter was inserted in 30% (6/20) of the observed births and in 19% (38/200) of the record reviews (Table 3). The SBAs didn’t ask women to pass urine regularly or before starting to push. When asked by the observers, the SBAs did not appear to be aware of the importance of an empty bladder in relation to uterine contractions and labour progress.

#### Active management of the third stage of labour

In 70% (14/20) of the observed births all aspects of active management of the third stage of labour (AMTSL) were performed correctly. The timing of the oxytocin administration varied from birth of the first shoulder to 5 minutes after birth. Generally, this was given at the correct time (6) or immediately after birth (9). Controlled cord traction was done correctly (in the right direction with the correct force) in 16 of the 20 women and uterine massage was performed well in 19 out of 20 births. In all observed women the time of birth of the placenta was recorded, but recorded information was not located in any of the record reviews. Examination of the placenta for completeness was done in 75% (15/20) of the observed deliveries, although in all files it was recorded as being done. In two of the observed deliveries the placenta appeared incomplete and a digital evacuation of the uterus was performed. Examination of the placenta for completeness was recorded in 46% (92/200) of the record reviews (Table 3).

#### Episiotomy and perineum care

There were four women (20%) who had episiotomy during observation of birth: one because of vacuum assisted birth, one because of shoulder dystocia, one because of fetal distress and one because of prolonged second stage. During almost every birth the SBA eased the perineum over the head with external perineal massage. In the record reviews 20% (39/200) of women had an episiotomy. In nulliparous women this was 49% (34/70). After the birth of the placenta all SBAs examined the perineum for tears and sutured if necessary. This was done correctly for the majority of women [85% (17/20)]. There were three incidents where the woman was clearly in pain from a SBA procedure and a lack of empathy was shown by the SBA. Two SBAs used a sponge forceps without addressing the woman and one forgot to give local anaesthesia before perineal repair, although this was later amended. On no occasion was a new pair of sterile gloves put on before starting to suture. Blood loss was measured in 18 observed deliveries by weighing the blood in the bucket underneath the birthing bed and adding any blood in pads and sarongs (traditional skirt worn by women). In two deliveries the blood loss was not measured but only estimated. In the record reviews only 4% (8/200) had an estimated amount written in the file as there was no place to routinely record it. In all these cases post-partum haemorrhage (PPH) had occurred and blood loss of >500 ml was written (Table 3).

#### Post-partum care and neonatal care

The majority of the women were transferred in less than one hour from the birth room to the post-partum area (the room next door) and this did not affect the monitoring of vital signs and blood loss post-partum, which was done at least once within two hours in 95% (19/20) of the observed women. Immediate neonatal care was done quickly and correctly on a clean surface. Apgar scores were assessed, the cord was clamped in a sterile manner and the baby’s weight was measured in all observed deliveries. In two cases oxygen was correctly given for nasal flaring and grunting to support breathing efforts of the baby. Formal monitoring of the baby in the first few hours after the birth only occurred in 30% (6/20) of the observations (Table 3). Often the baby was not checked again until the next day.

#### Complications

Most of the observed births occurred without complications [70% (14/20)]. In the remaining six women the complications included one vacuum assisted birth, one shoulder dystocia, one case of mild pre-eclampsia and three PPHs in which the estimated blood loss ranged from 520 to 1470 ml. One woman received two units of fresh blood at the SMRU clinic, one immediately after the birth and one two days later. In these cases, the SBAs were observed to be slow to recognize severity and take action. The SBAs followed the protocol, but it sometimes took considerable time from identification of the problem to providing the corrective measure; such as inserting an intravenous line for a fluid bolus or inserting a urine catheter to measure output. In one case of PPH due to suspected retained placenta tissue the delay was seven hours before the SBA discussed with the doctor. In the record reviews 5.5% (11/200) women had PPH recorded but only eight of these mentioned a blood loss of >500 ml in the notes. Post-partum infection was observed in 10% (2/20) women and recorded in 5% (10/200) record reviews (Table 3).

There were 26 records identified with prolonged labour and suspected obstructed labour and in all cases the SBAs used the alert and action line on the partograph. Artificial rupture of membranes (AROM) when indicated was not delayed [100% (16/16)] as 38.5% (10/26) already had spontaneous rupture of membranes. In 23% (6/26) oxytocin augmentation was not done while it was indicated. In the 30% (6/20) of cases that received oxytocin augmentation it was started with delay. Overall 65% (17/26) had a normal vaginal birth, 12% (3/26) had a vacuum assisted birth and 23% (6/26) had a caesarean section in the nearest Thai hospital. All 26 cases resulted in a live born neonate none of whom required assistance in initial resuscitation.

There were nine records identified with pre-eclampsia and eclampsia using SMNA criteria: two women had eclampsia, one had severe pre-eclampsia and the other six had mild pre-eclampsia. All eclampsia and severe pre-eclampsia cases received timely administration of anticonvulsants (magnesium sulphate) and antihypertensive drugs (hydralazine, nifedipine and/or methyldopa). Of the mild pre-eclampsia cases two did not receive any antihypertensive drugs. Proteinuria was checked in all cases, but intra-partum monitoring was suboptimal as in 56% (5/9) either maternal blood pressure or fetal heart beat were not checked hourly.

## Discussion

The components of the adapted WHO SMNA tool: facility assessment, SBA interviews, observations of labour and child birth and record reviews; were complementary and useful to provide a detailed synopsis of the strengths and weakness of the quality of care by SBAs in Mae La refugee camp. The most powerful part of the tool in this setting was the direct observation of SBAs providing care in labour and child birth. Incidents of substandard care and harmful practices conferred with previously reported studies in developing countries [22-24]. Importantly these incidents were observed during labour and child birth and could not be gleaned from record reviews or SBA interviews.

Infections rates of 15% of observed labour and births and 5% on record reviews were consistent with random and infrequent hand washing. Maternal and newborn health organizations have been promoting clean birth practices as the ‘six cleans’: clean hands of the SBA, clean perineum of the mother, a clean birth surface, a clean cord cutting instrument, a clean cord clamping device and clean cord care [25]. To their credit the observations of labour and birth showed full adherence of the SBAs to the last five ‘cleans’, but hand washing was substandard and strategies to improve compliance have been challenging even in resource rich settings [26]. High bladder catheterization rates can be harmful [27] and were confirmed in both record reviews and observations. Communication and kindness which can only be observed were also identified as an area for attention [28]. The low proportion of monitoring the blood pressure in labour differs because the SBAs measured the blood pressure two hourly according to the SMRU guideline which was based on a low staff to patient ratio, a particular consideration for quality of care in low resource settings [29]. Records were generally complete and consistent with observations of labour and child birth. Actively measuring and estimating the blood loss was introduced just prior to the observation period for this assessment, together with an obligatory item on the patient records to fill in the estimated blood loss, and this explains the major difference between the record reviews and the observations.

PPH is the most common complication in the SMRU birth room and SBAs always applied active management of the third stage of labour. In 20% of cases controlled cord traction was incorrectly applied which could be harmful and is pertinent following the recent publication that reported no benefit from this procedure [30]. SBA interviews and observation of child birth also identified differences between practice and knowledge: for example there was high awareness of the importance of the placenta being complete but carelessness was observed in confirming this. One of the most important observations was the difficulty in recognizing the severity of PPH and taking affirmative action. This will lead to delay and in the situation of PPH delay is associated with maternal mortality. Teaching speed for EmONC in the birth room is a challenge [31] and identifying how SBAs best acquire this skill has not been reported. In addition the SBAs in Mae La refugee camp who staff the birth centre are the people available from the population and this has its own constraints. In general record completion and competence in routine work such as correct use of the WHO partograph, monitoring the fetal heart beat in labour, and active management of the third stage of labour, were good, which is often difficult to achieve [22-24,32]. However when the situation deviated from normal, for example when the vital signs indicated that PPH was severe, or that the fetal heart beat was worrisome; a lack of depth of understanding started to emerge. Organizations cannot replace the vital years of schooling that is missing for an entire generation in protracted conflict situations so the pedagogy of how best to work within these constraints has to be developed [33].

Agreement between observed labours and births and record reviews for episiotomy rates was reassuring. In nulliparous women the rate of 49% falls between the average of routine episiotomy at 75% and of restrictive episiotomy at 28% [34]. During direct observation episiotomy was only applied for specified indications following the restrictive guidelines but it is unknown what influence the presence of the observers may have had. Support during labour was adequate and there is ample evidence of the beneficial influence of a supportive companion of the woman’s choice on her satisfaction and many aspects of the birth process [35,36]. Immediate neonatal care and initial assessment of the newborn was clean, efficient and appropriate. Postnatal monitoring was inadequate and needs improvement [7]. The low rate of antibiotic eye drops is correct according to local guidelines as observed STI and HIV rates are very low in the area [37]. Skin-to-skin contact, timing of first contact with the mother and timing of first breastfeeding have not been included in the observations, as these have been reported previously [38].

The birth unit did not suffer from lack of necessary equipment, medicines and infrastructure which were all available according to the facility assessment, contrary to the supply shortages previously described in nearby areas [12]. This creates an enabling environment for the SBAs to perform their tasks, which is important for the motivation of the staff and the perceived quality of the health care facility by the patients [39].

This study has several limitations. As the data collection was done during a short six week interval some less common problems may not have had an opportunity to be observed, nevertheless 20 observed labour and births provided plenty of points for quality improvement. Record reviews occurred prior (three months) to the observations and the interviews. Some aspects of practice were already in the process of improving by the time the observations were carried out, which explains some differences between record review and observation data: e.g. systematically recording post-partum blood loss. For the observations of labour and birth the language barrier may have been a limitation and some aspects of care may have been overlooked or interpreted or reported incorrectly. The observations themselves could have introduced bias in performances, such as the SBAs trying to do their very best. This Hawthorne effect where people may modify their behaviour because of being observed [40] has been demonstrated to influence the time interval until cesarean section [41]. The ability to generalize this study may be limited to birth centres where SBAs with similar background, level and responsibilities are the primary provider. However a recent systematic review and meta-analysis suggests that the level of the heath care provider is not significant in the effectiveness of care provided [29]. Lastly the initial data were collected in 2008 and while changes have taken place in the past six years these will be presented in a future publication to complete the audit cycle.

The WHO SMNA has been a useful tool to assess the quality of intrapartum care provided by SBAs in Mae La refugee camp. Adapting the tool for the setting and allowing local staff to apply the tool minimized the costs associated with the assessment. The strength of the tool lies in the combination of record reviews and direct observation and this has been reported in similar investigations using direct clinical observations [41,42].

## Conclusions

In the SMRU birth centre in Mae La refugee camp SBAs were able to comply with evidence-based guidelines but support to improve quality of care in specific areas is required. The structure of the WHO SMNA allowed significant insights into practice particularly through direct observation identifying a clear pathway for quality improvement.

## Additional file

Additional file 1:
**Adapted SMNA survey forms.**

## Abbreviations

AMTSL: Active management of the third stage of labour
ANC: Antenatal care
AROM: Artificial rupture of membranes
EmONC: Emergency obstetric and neonatal care
NGO: Non-governmental organization
NVD: Normal vaginal delivery
IMPAC: Integrated management of pregnancy and childbirth
PMTCT: Prevention of mother-to-child transmission of HIV
PPH: Post-partum haemorrhage
PU-AMI: Première Urgence–Aide Médicale International
SBA: Skilled birth attendant
SMNA: Safe motherhood needs assessment
SMRU: Shoklo Malaria Research Unit
TBA: Traditional birth attendant
WHO: World Health Organization
