# Supplementary material for: Quality of intrapartum care by skilled birth attendants in a refugee clinic on the Thai-Myanmar border: a survey using WHO Safe Motherhood Needs Assessment
Source: BMC Pregnancy Childbirth. 2015 Feb 5;15:17. doi: 10.1186/s12884-015-0444-0 (PMC4332741; doi:10.1186/s12884-015-0444-0)
Supplement: Additional file 1: — Adapted SMNA survey forms. [file 12884_2015_444_MOESM1_ESM.pdf]

Data entry sequence number	Data entry initials:

Antenatal Record Review (weekly ANC CRF + Risk Factor Sheet)

Date today:		ANC code:		Surveyor code:	

Please see instructions in Surveyor's Manual.

{ANR1}	HOW MANY ANTENATAL CARE (ANC) VISITS ARE RECORDED ON THE CARD?<br>*Count the number of visits on the record and enter total*	
{ANR2}	WHAT IS THE GESTATIONAL AGE IN WEEKS AT FIRST ANC VISIT AS RECORDED ON THE CARD?<br>*Enter number of weeks. If not known, enter 0*	
{ANR3}	HOW MANY TIMES WAS THE BLOOD PRESSURE (BP) MEASURED AND RECORDED ON THE CARD?<br>*Enter the **number** of recordings*<br>***Do not enter actual BP readings!***<br>*If none, enter 0*	
{ANR4}	HOW MANY TIMES WAS THE PROTEINURIA MEASURED AND RECORDED ON THE CARD?<br>*Enter the **number** of recordings*<br>***Do not enter actual test results!***<br>*If none, enter 0*	
{ANR5}	HAS GRAVIDA (NUMBER OF PREGNANCIES INCLUDING PRESENT PREGNANCY) BEEN RECORDED ON THE RECORD?<br>*Tick box*	1 Yes<br>0 No
{ANR6}	ARE THE RESULTS OF A HAEMATOCRIT TEST RECORDED ON THE RECORD?<br>*Tick box*	1 Yes<br>0 No
{ANR7}	ARE THE RESULTS OF A SYPHILIS TEST RECORDED ON THE RECORD?<br>*Tick box*	1 Yes<br>0 No
{ANR8}	WAS SUPPLEMENTATION WITH IRON/FOLIC ACID RECORDED ON THE RECORD?<br>*Tick box*	1 Yes<br>0 No
{ANR9}	ARE THE RESULTS OF A MALARIA TEST RECORDED ON THE RECORD?<br>*Tick box*	1 Yes<br>0 No
{ANR10}	IF THE MALARIA TEST WAS POSITIVE, WAS THE PROVISION OF MALARIA TREATMENT RECORDED ON THE RECORD?<br>*Tick box*	1 Yes<br>0 No
{ANR11}	ARE RISKFACTORS RECORDED ON THE RECORD?<br>*Tick box*	1 Yes<br>0 No
ANR12}	IS A PMTCT TEST RECORDED ON THE RECORD?<br>*Tick box*	1 Yes<br>0 No
ANR13}	IS A PMTCT TEST WAS DONE, IS POST-TEST-COUNSELING RECORDED ON THE RECORD?<br>*Tick box*	1 Yes<br>0 No

Data entry sequence number:	Data entry initials:

Complicated delivery (pre-)eclampsia record review (whole file including IPD chart)

Date today:		ANC code:		Surveyor code:	

Please see instructions Surveyor's Manual. Select the first records on the criteria outlined below:

eclamptic fits are recorded
OR
diastolic blood pressure is greater than or equal to 100 mm Hg (for example, 140/100 mm Hg)

Record the following information from the record:

{CDE1}	IS THE DIASTOLIC BLOOD PRESSURE RECORDED ON THE CARD GREATER THAN OR EQUAL TO 100 (FOR EXAMPLE, 140/100, ETC.)? *Tick box*	1 Yes<br>0 No
{CDE2}	WAS THE ADMINISTRATION OF ANTIHYPERTENSIVE MEDICATION RECORDED (FOR EXAMPLE, ALDOMET, NIFEDIPINE OR HYDRALAZINE)? *Tick box*	1 Yes<br>0 No
{CDE3}	IS ECLAMPSIA OR ARE ECLAMPTIC FITS RECORDED ON THE CARD? *Tick box*	1 Yes<br>0 No
{CDE4}	WAS THE ADMINISTRATION OF SEDATIVE OR ANTICONVULSIVE MEDICATION RECORDED (FOR EXAMPLE MAGNESIUM SULFATE OR DIAZEPAM)? *Tick box*	1 Yes<br>0 No
{CDE5}	WAS THE BLOOD PRESSURE CHECKED AND RECORDED AT LEAST HOURLY? *Tick box*	1 Yes<br>0 No
{CDE6}	WAS THE FETAL HEART BEAT CHECKED AND RECORDED AT LEAST HOURLY? *Tick box*	1 Yes<br>0 No
{CDE7}	WAS PROTEINURIA CHECKED AND RECORDED? *Tick box*	1 Yes<br>0 No
{CDE8}	WERE SYMPTOMS AND (DANGER) SIGNS CHECKED AND RECORDED (FOR EXAMPLE HEADACHE, BLURRED VISION, EPIGASTRIC PAIN, OEDEMA OR INCREASED REFLEXES)? *Tick box*	1 Yes<br>0 No

Data entry sequence number:	Data entry initials:

Complicated delivery (obstructed labour) record review (Partogram + Outcome Sheet)

Date today:		ANC code:		Surveyor code:	

Please see instructions Surveyor's Manual.

*Select the **first records** that meet the selection criteria outlined below:*

descent is static for three hours or more
OR
strong contractions with no progress for three hours

Exclude multiple birth (twin, triplet etc.)
Exclude non-cephalic birth (breech, shoulder first, etc.)

Record the following information from the record:

{CDO1}	WAS THE DESCENT OF THE HEAD STATIC (NO PROGRESS FOR THREE HOURS OR MORE) *Tick box*	1 Yes<br>0 No
{CDO2}	WERE STRONG CONTRACTIONS RECORDED FOR MORE THAN THREE HOURS WITHOUT DESCENT OF THE HEAD? *Tick box*	1 Yes<br>0 No
{CDO3}	WHAT IS THE RECORDED CONDITION OF THE BABY AT BIRTH? *Tick box*	1 Stillbirth<br>2 Live birth, not good condition (Apgar score 6 or less or equivalent)<br>3 Live birth, good condition (Apgar 7-10 or equivalent)<br>4 Live birth, condition not recorded<br>0 Not recorded
{CDO4}	WHAT IS THE RECORDED MODE OF DELIVERY? *Tick box*	1 Normal vaginal delivery<br>2 Vacuum<br>3 Forceps<br>4 Caesarean section<br>0 Not recorded
{CDO5}	WHEN ACTION LINE WAS CROSSED WHAT ACTION WAS TAKEN? *Tick box*	1 AROM<br>2 augmentation with syntocinon<br>3 referral<br>4 action line was not reached<br>0 no action
{CDO6}	WAS AROM DONE? *Tick box*	1 yes, in time<br>2 yes, with delay<br>3 already SROM<br>0 not indicated
{CDO7}	WAS AUGMENTATION WITH SYNTOCINON DONE? *Tick box*	1 yes, in time<br>2 yes, with delay<br>0 not indicated / contra-indicated
{CDO8}	IN CASE OF CAESAREAN SECTION, HOW MANY HOURS ELAPSED PAST THE ACTION LINE (TO THE RIGHT OF THE LINE) BEFORE REFERRAL TOOK PLACE? *Enter number of hours* *If referral took place before the action line, enter hours as negative (e.g. "-1")*	

Data entry sequence number:	Data entry initials:

Facility Management

Date today:				Surveyor code:	

After initially meeting with the midwife in charge to ask questions on pages 1-3, explain that you would like to be shown the clinic to gather the remaining information.

{FAC1A}	WHAT IS THE ESTIMATED POPULATION SERVED BY SMRU?<br>*Enter population; if not known enter 0*		
{FAC1B}	HOW MANY PATIENTS FIT IN THE CLINIC (INCLUDING OBSTETRIC DEPARTMENT)?<br>*Enter number*		
{FAC1C}	HOW MANY PATIENTS FIT IN THE OBSTETRIC DEPARTMENT?<br>*Enter number*		
{FAC1D}	OF ALL PREGNANT WOMEN IN MAE LA HOW MANY ATTEND ANC?<br>*Enter percentage; if not known do not calculate, enter 0*	%	
{FAC1E}	OF ALL WOMEN THAT ATTEND ANC HOW MANY DELIVER IN SMRU?<br>*Enter percentage; if not known do not calculate, enter 0*	%	
	AT SMRU HOW MANY POSITIONS ARE THERE FOR:	Enter number of positions	
		Midwives	Nurses
{FAC2A}	QUALIFIED MIDWIVES AND NURSES (WITH OFFICIAL QUALIFICATION)		
		Senior	Junior
{FAC2B}	SENIOR AND JUNIOR MIDWIVES		
		Midwife assistant	Volunteer
{FAC2C}	MIDWIFE ASSISTANTS AND VOLUNTEERS		
		Obstetrics	Others
{FAC2D}	PHYSICIANS (BOTH OBSTETRICS AND OTHER SPECIALITIES)		

	WHICH OF THE FOLLOWING SERVICES HAVE BEEN PROVIDED WITHIN THE PAST **SIX MONTHS** AT SMRU?<br>*Ask about each service separately*	*Tick one box for each item*	
{FAC3A}	ANTENATAL CARE	1 Yes	0 No
{FAC3B}	TETANUS TOXOID IMMUNIZATION	1 Yes	0 No
{FAC3C}	MANAGEMENT OF SEXUALLY TRANSMITTED INFECTIONS	1 Yes	0 No
{FAC3D}	NORMAL DELIVERY CARE	1 Yes	0 No
{FAC3E}	VACUUM DELIVERY	1 Yes	0 No
{FAC3F}	CAN FAMILY STAY WITH MOTHER AND BABY AFTER DELIVERY	1 Yes	0 No
{FAC3G}	POSTPARTUM CHECK UP	1 Yes	0 No
{FAC3H}	FAMILY PLANNING SERVICES	1 Yes	0 No
{FAC3J}	ABORTION ON REQUEST	1 Yes	0 No
{FAC3K}	BLOOD TRANSFUSION	1 Yes	0 No
{FAC3L}	CAESAREAN SECTION	1 Yes	0 No

	WHICH OF THE FOLLOWING COMPLICATIONS HAVE HAPPENED AND HAVE BEEN MANAGED AT SMRU WITHIN THE PAST **SIX MONTHS?**<br><br>*Ask about each service separately*	*Tick one box for each item*	
{FAC4A}	SEVERE ANAEMIA	☐ 1 Yes	☐ 0 No
{FAC4B}	ANTEPARTUM HAEMORRHAGE	☐ 1 Yes	☐ 0 No
{FAC4C}	PRE-ECLAMPSIA	☐ 1 Yes	☐ 0 No
{FAC4D}	ECLAMPSIA	☐ 1 Yes	☐ 0 No
{FAC4E}	POSTPARTUM HAEMORRHAGE	☐ 1 Yes	☐ 0 No
{FAC4F}	ABORTION COMPLICATIONS	☐ 1 Yes	☐ 0 No
{FAC4G}	RETAINED PLACENTA	☐ 1 Yes	☐ 0 No
{FAC4H}	BREECH PRESENTATION/DELIVERY	☐ 1 Yes	☐ 0 No
{FAC4J}	SEPSIS	☐ 1 Yes	☐ 0 No
{FAC4K}	ECTOPIC PREGNANCY	☐ 1 Yes	☐ 0 No
{FAC4L}	RUPTURED UTERUS	☐ 1 Yes	☐ 0 No

	ARE THE FOLLOWING SERVICES PROVIDED EVERY DAY THAT SMRU IS OPEN?<br><br>*Ask about each service separately*	*Tick one box for each item*	
{FAC5A}	ANTENATAL CARE	☐ 1 Yes	☐ 0 No
{FAC5B}	FAMILY PLANNING SERVICES	☐ 1 Yes	☐ 0 No
{FAC5C}	CHILD HEALTH CARE	☐ 1 Yes	☐ 0 No

	WHICH OF THE FOLLOWING LABORATORY SERVICES HAVE BEEN PROVIDED AT SMRU WITHIN THE **PAST WEEK?**<br>*Ask about each service separately*	*Tick one box for each item*	
{FAC6A}	SYPHILIS TESTING	☐ 1 Yes	☐ 0 No
{FAC6B}	HAEMATOCRIT MEASUREMENT	☐ 1 Yes	☐ 0 No
{FAC6C}	URINE TESTING - PROTEIN MEASUREMENT	☐ 1 Yes	☐ 0 No
{FAC6D}	PMTCT	☐ 1 Yes	☐ 0 No
{FAC6E}	MALARIA TESTING	☐ 1 Yes	☐ 0 No

Emergency services and referral

{FAC7A}	IS STAFF AVAILABLE FOR DELIVERIES AT NIGHT AND AT WEEKENDS?	☐ 1 Yes	☐ 0 No
{FAC7B}	CAN YOU CALL THE DOCTOR FOR CARE OF COMPLICATED DELIVERIES AT NIGHT AND AT WEEKENDS?	☐ 1 Yes	☐ 0 No
{FAC7C}	CAN YOU SEND TO MAE SOT HOSPITAL FOR CAESAREAN SECTION AT NIGHT AND AT WEEKENDS?	☐ 1 Yes	☐ 0 No
{FAC7D}	IS A CAR ALWAYS AVAILABLE FOR AN OBSTETRIC EMERGENCY REFERRAL?	☐ 1 Yes	☐ 0 No
{FAC7E}	ONCE YOU DECIDE TO REFER AN OBSTETRIC EMERGENCY CASE, ABOUT HOW LONG DOES IT TAKE FOR HER TO ARRIVE AT MAE SOT HOSPITAL AND RECEIVE CARE?<br>*Enter number of hours*<br>*Enter 0 for referral centre/hospital*		
{FAC7F}	HOW FAR IS MAE SOT HOSPITAL, IN KILOMETRES?<br>*Enter number of kilometres, one way*<br>*Enter 0 for referral centre/hospital*		

{FAC7G}	WHO, APART FROM THE DRIVER, USUALLY GOES WITH AN EMERGENCY REFERRAL PATIENT TO THE HOSPITAL?<br>*Tick one box*	☐ 0 Patient is not accompanied (driver only)<br>☐ 1 Nurse/Midwife<br>☐ 2 Medic<br>☐ 3 Family member<br>☐ 4 Other - specify

Infrastructure and equipment

	WHICH OF THE FOLLOWING ITEMS ARE AVAILABLE AND IN SATISFACTORY CONDITION?<br><br>*Ask about each item separately. Code as unsatisfactory items which in your judgement are not functional, have missing parts, are unhygienic, or otherwise sub-standard. Be sure to* ***look*** *at each item.*	*Enter correct number below*<br><br>0 = Not available<br>1 = Available but not satisfactory<br>2 = Available and satisfactory<br>9 = Not applicable for this facility
{FAC8A}	EXAMINATION ROOM OR AREA PROVIDING CLIENT PRIVACY (ROOM FOR SCREENING, COUNSELLING AND EXAMINATION)	
{FAC8B}	TABLE AND STOOL FOR GYNAECOLOGICAL EXAMINATIONS	
{FAC8C}	STORAGE AREA OR CUPBOARD FOR DRUGS AND OTHER SUPPLIES	
{FAC8D}	TOILET FACILITIES OR LATRINE	
{FAC8E}	DELIVERY OR LABOUR ROOM WITH BED AND LIGHTING	
{FAC8F}	REFRIGERATOR	
{FAC8G}	WATER SUPPLY	
{FAC8H}	TELEPHONE OR RADIO TRANSMITTER	
{FAC8J}	AMBULANCE OR VEHICLE TO REFER AN OBSTETRIC EMERGENCY	
	WHICH OF THE FOLLOWING ITEMS ARE AVAILABLE AND IN SATISFACTORY CONDITION?<br><br>*Ask about each item separately. Code as unsatisfactory items which in your judgement are not functional, have missing parts, are unhygienic, or otherwise sub-standard. Be sure to* ***look*** *at each item.*	*Enter correct number below*<br><br>0 = Not available<br>1 = Available but not satisfactory<br>2 = Available and satisfactory<br>9 = Not applicable for this facility
	**Registers**	
{FAC9A}	GUIDELINES/PROTOCOLS FOR MATERNAL HEALTH CARE	
{FAC9B}	DELIVERY LOG BOOK	
{FAC9C}	ANTENATAL LOG BOOK	
{FAC9D}	FAMILY PLANNING LOG BOOK	
	**Basic equipment**	
{FAC10A}	BLOOD PRESSURE APPARATUS (SPHYGMOMANOMETER)	
{FAC10B}	STETHOSCOPE	
{FAC10C}	INFANT WEIGHING SCALE	
{FAC10D}	FETAL STETHOSCOPE / DOPPLER	
{FAC10E}	STERILIZER	
{FAC10F}	THERMOMETER	
{FAC10G}	MANUAL VACUUM ASPIRATOR (MVA)	
{FAC10H}	PROTECTIVE CLOTHING (SHOES, APRONS)	
{FAC10J}	SPECULUM (VARIOUS SIZES)	
{FAC10K}	VACUUM	
{FAC10L}	FORCEPS	
{FAC10M}	EMERGENCY BOX (COMPLETE)	
	**Absolute minimum equipment for delivery**	
{FAC11A}	SCISSORS	
{FAC11B}	SUTURE MATERIAL	
{FAC11C}	NEEDLE HOLDER	

	**Absolute minimum for care of neonate**	
{FAC12A}	TOWEL TO DRY BABY	
{FAC12B}	BLANKET TO WRAP BABY	
{FAC12C}	AMBU-BAG AND MASK FOR NEONATAL RESUSCITATION	
	**Educational materials...**	
{FAC13A}	... ON DANGER SIGNS OF COMPLICATIONS IN PREGNANCY	
{FAC13B}	... ON POSTPARTUM OR NEWBORN CARE OR BREAST-FEEDING	
{FAC13C}	... ON FAMILY PLANNING	
{FAC13D}	... ON SEXUALLY TRANSMITTED INFECTIONS / HIV/AIDS	
{FAC13E}	...ON ANTENATAL NUTRITION OR ANAEMIA	

{FAC14A}	IS ALL EQUIPMENT THAT YOU REQUIRE FOR FAMILY PLANNING/CONTRACEPTION AVAILABLE? *If equipment is not available or unsatisfactory, please specify below:*	1 Yes	0 No
{FAC14B}	IS ALL EQUIPMENT THAT YOU REQUIRE FOR CAESAREAN SECTION AVAILABLE? *If equipment is not available or unsatisfactory, please specify below:*	1 Yes	0 No

Essential drugs and consumable supplies

	For essential drugs and consumable supplies, it is sufficient to look for and see that a particular item is available, regardless of condition or expiration date.	*Enter correct number below*<br><br>0 = Not seen at facility<br>1 = Seen at facility<br>9 = Not applicable for this facility
{FAC15A}	GLOVES	
{FAC15B}	DISPOSABLE SYRINGES AND NEEDLES	
{FAC15C}	IV KIT	
{FAC15D}	BLANK PARTOGRAMS	
{FAC15E}	BLANK ANTENATAL CRF'S AND DELIVERY OUTCOME SHEETS	
{FAC15F}	CORD TIES	
{FAC15G}	BLOOD GIVING SETS	
{FAC15H}	SYPHILIS TEST KITS	
{FAC15J}	URINE DIP STICK / PROTEINURIA TEST SUPPLIES	
	**Anaesthetics: general**	
{FAC16B}	DIAZEPAM (INJECTION)	
{FAC16C}	KETAMINE (INJECTION)	
	**Anaesthetics: local**	
{FAC17A}	XYLOCAINE 2% OR OTHER	
	**Analgesics**	
{FAC18A}	PETHIDINE	
	**Anti-infective drugs: antibacterial (mother)**	
{FAC19A}	AMPICILLIN (CAPSULES OR INJECTION)	
{FAC19B}	BENZATHINE BENZYLPENICILLIN **OR** PROCAINE BENZYLPENICILLIN (INJECTIONS)	

{FAC19C}	CEFTRIAXONE (INJECTION) **OR** CIPROFLOXACIN (CAPSULE)	
{FAC19D}	GENTAMICIN (INJECTION)	
{FAC19E}	KANAMYCIN (INJECTION)	
{FAC19F}	CO-TRIMOXAZOLE (TABLETS)	
	For essential drugs and consumable supplies, it is sufficient to look for and see that a particular item is available, regardless of condition or expiration date.	Enter correct number below<br><br>0 = Not seen at facility<br>1 = Seen at facility<br>9 = Not applicable for this facility
	**Anti-infective drugs: antibacterial (neonate)**	
{FAC20A}	TETRACYCLINE (OINTMENT) **OR** CHLOORAMPHENICOL (EYE DROPS)	
	**Anti-infective drugs: antimalarials**	
{FAC21A}	ARTESUNATE	
{FAC21B}	QUININE (INJECTION) **OR** CHLOROQUINE (INJECTION)	
	**Antianaemia drugs**	
{FAC22A}	FERROUS SALT+FOLIC ACID (IN COMBINED FORM OR SEPARATELY)	
	**Antihypertensive drugs**	
{FAC23A}	ALDOMET **OR** PROPRANOLOL **OR** NIFEDIPINE	
{FAC23B}	HYDRALAZINE (INJECTION)	
	**Anticonvulsive drugs**	
{FAC24A}	MAGNESIUM SULFATE (INJECTION) **OR** DIAZEPAM (INJECTION)	
	**Contraceptives**	
{FAC25A}	ORAL CONTRACEPTIVES (ANY TYPE)	
{FAC25B}	INJECTABLE CONTRACEPTIVES (ANY TYPE)	
{FAC25C}	CONDOMS	
{FAC25D}	IUCDS/IUDS	
	**Immunologicals: Vaccines**	
{FAC26A}	TETANUS TOXOID (INJECTION) STORED IN REFRIGERATOR	
{FAC26B}	BCG VACCINE (INJECTION)	
	**Oxytocics**	
{FAC27A}	METHERGYNE (INJECTION) **OR** OXYTOCIN (INJECTION)	
	**Disinfectants and antiseptics**	
{FAC28A}	CHLORHEXIDINE **OR** ALCOHOL **OR** POVIDONE	
	**Intravenous solutions**	
{FAC29A}	NSS **OR** RINGER'S LACTATE **OR** D5W	

Delivery registry and presentation of maternal complications

*Ask to see the delivery log book. Tally information on the number of cases of various conditions from the delivery registry or other relevant records **for the past 12 months**. Space is provided under each condition for tallying the number of cases. After completion, enter numerical totals in the right hand column. Exclude cases in which the baby was born before arrival at the facility.*

	Use space below for tally	*Enter number below*
{FAC30A}	TOTAL NUMBER OF BIRTHS (FOR PAST 12 MONTHS)<br>*(Count tally here e.g. IIII)*	

{FAC30B}	INSTRUMENTAL DELIVERIES (VACUUM OR FORCEPS)	
{FAC30C}	ABNORMAL VAGINAL DELIVERIES (BREECH, FACE, SHOULDER DELIVERIES)	
{FAC30D}	CAESAREAN SECTIONS	
{FAC30E}	MATERNAL DEATHS	
{FAC30F}	STILLBIRTHS	
{FAC30G}	NEONATAL DEATHS	

Family planning register

Ask to see the family planning register or log book. Tally information on the number of users of various contraceptive methods ***for the past 3 months****. For sterilization, it might be necessary to look at the operating theatre register. Space is provided under each family planning type for tallying the number of clients. After completion, enter numerical totals in the right-hand column.*

	Use space below for tally	*Enter number below*
{FAC31H}	STERILIZATION: FEMALE	

Monitoring and evaluation

{FAC32A}	IS INFORMATION ON MATERNAL DEATHS ROUTINELY REPORTED BY MAE LA TO MAE SOT OFFICE? *Tick box* *Tick box*	☐ 2 Always<br>☐ 1 Sometimes<br>☐ 0 Never
{FAC32B}	IS INFORMATION ON MATERNAL DEATHS ROUTINELY REPORTED BY SMRU TO HEALTH INFORMATION SYSTEM? *Tick box*	☐ 2 Always<br>☐ 1 Sometimes<br>☐ 0 Never
{FAC32C}	ARE STANDARD ANTENATAL CRF'S USED IN MAE LA? *Tick box*	☐ 2 Always<br>☐ 1 Sometimes<br>☐ 0 Never
{FAC32D}	ARE STANDARD PARTOGRAMS AND OUTCOMES SHEETS USED MAE LA? *Tick box*	☐ 2 Always<br>☐ 1 Sometimes<br>☐ 0 Never
{FAC32E}	ARE CONFIDENTIAL ENQUIRIES OR AUDITS ROUTINELY DONE FOLLOWING MATERNAL DEATHS? *Tick box*	☐ 2 Always<br>☐ 1 Sometimes<br>☐ 0 Never

Data entry sequence number:	Data entry initials:

Normal Delivery Record Review (Whole file)

Date today:		ANC code:		Surveyor code:	

Please see instructions in Surveyor's Manual.

{NDR1}	CALCULATE THE NUMBER OF HOURS FROM START PARTOGROM UNTIL TIME OF DELIVERY. FOR EXAMPLE, IF THE PARTOGRAM WAS STARTED AT 8:00 AM AND SHE DELIVERED AT 6:00 PM, ENTER "10"<br>*Record number of hours*<br>*Round fractions down (i.e. record 2:30 as 2 hours)*<br>*If delivery occurred less than one hour after admission, enter 1*<br>*If not available, enter 0*	
{NDR2}	HOW MANY VAGINAL EXAMINATIONS ARE RECORDED ON THE CARD?<br>*Enter the number of examinations recorded. If none, enter 0*	
{NDR3}	HOW MANY FETAL HEARTBEAT READINGS ARE RECORDED ON THE CARD?<br>*Enter the **number** of recordings*<br>***Do not enter actual readings!***<br>*If none, enter 0*	
{NDR4}	HOW MANY TIMES WAS THE BLOOD PRESSURE (BP) MEASURED AND RECORDED ON THE CARD?<br>*Enter the **number** of recordings*<br>***Do not enter actual BP readings!***<br>*If none, enter 0*	
{NDR5}	IS THE BIRTH WEIGHT OF THE BABY RECORDED ON THE CARD?<br>*Tick box*	1 Yes<br>0 No
{NDR6}	IS ANY ASSESSMENT OF THE CONDITION OF THE BABY RECORDED ON THE CARD (E.G. APGAR SCORE)?<br>***Do not enter actual condition or Apgar score!***<br>*Tick box*	1 Yes<br>0 No
{NDR7}	IS ANY ANTENATAL CARE RECORDED ON THE CARD?<br>*Tick box*	1 Yes<br>0 No
{NDR8}	WHAT IS RECORDED AS GRAVIDA (NUMBER OF PREGNANCIES INCLUDING PRESENT PREGNANCY) ON THE RECORD?<br>*Enter the number recorded behind 'G'.*	
{NDR9}	WHAT IS RECORDED AS PARA (NUMBER OF CHILDREN EXCLUDING PRESENT PREGNANCY) ON THE RECORD?<br>*Enter the number recorded behind 'P'.*	
{NDR10}	WHAT IS RECORDED FOR PERINEUM ON THE RECORD<br>*Tick box*	1 intact<br>2 old tear & intact<br>3 minor tear (no repair)<br>4 tear & repair<br>5 epi & repair<br>6 other…
{NDR11}	HAS A URINE CATHETER BEEN INSERTED DURING LABOUR?<br>*Tick box*	1 Yes<br>0 No
{NDR12}	DID POSTPARTUM HEAMORRHAGE OCCUR AFTER BIRTH?<br>*Tick box*	1 Yes<br>0 No

{NDR13}	HOW MUCH WAS THE ESTIMATED BLOODLOSS?<br>*Enter the amount in cc.*	
{NDR14}	WAS PLACENTA DELIVERY TIME RECORDED ON THE RECORD?<br>*Tick box*	1 Yes<br>0 No
{NDR15}	WAS THE PLACENTA COMPLETE?<br>*Tick box*	1 Yes<br>0 No<br>9 Not recorded
{NDR16}	DID ANY POSTPARTUM INFECTION OCCUR?<br>*Tick box*	1 infected tear<br>2 infected epi<br>3 endometritis<br>4 UTI<br>5 mastitis<br>6 puerperal sepsis<br>7 other…<br>0 no
{NDR17}	DID ANY OPERATION TAKE PLACE AFTER THIS DELIVERY?<br>*Tick box*	1 manual placenta removal<br>2 digital evacuation<br>3 digital evacuation & curettage<br>4 curettage<br>5 sponge forceps<br>6 other…<br>0 no

Data entry sequence number:	Data entry initials:

Interview with midwife or maternity nurse

Date today:				Surveyor code:	

Please see instructions in Surveyor's Manual.

{NMW1}	WHEN WAS THE LAST TIME THAT YOU DID A DELIVERY?<br>*Tick one best response*	0 Never<br>1 In the past week<br>2 In the past month<br>3 In the past 6 months<br>4 6 months ago or longer
{NMW2}	AFTER A NORMAL DELIVERY, DO YOU ASK A CLIENT TO RETURN? IF YES, WHEN SHOULD SHE RETURN?<br>*Tick one best response*	0 Never / do not ask<br>1 During first week<br>2 During first six weeks<br>3 Only if she is ill / in case of problem<br>4 For study<br>9 Other - specify:
{NMW3}	WHEN WAS THE LAST TIME THAT YOU SAW A WOMAN WITH POSTPARTUM HAEMORRHAGE?<br>*Tick one best response*	0 Never<br>1 In the past week<br>2 In the past month<br>3 In the past 6 months<br>4 6 months or longer
{NMW4}	WHEN WAS THE LAST TIME THAT YOU SAW A WOMAN WITH OBSTRUCTED LABOUR?<br>*Tick one best response*	0 Never<br>1 In the past week<br>2 In the past month<br>3 In the past 6 months<br>4 6 months ago or longer
{NMW5}	WHEN WAS THE LAST TIME THAT YOU SAW A WOMAN WITH PUERPERAL SEPSIS?<br>*Tick one best response*	0 Never<br>1 In the past week<br>2 In the past month<br>3 In the past 6 months<br>4 6 months ago or longer
{NMW6}	WHEN WAS THE LAST TIME THAT YOU SAW A WOMAN WITH (PRE-)ECLAMPSIA?<br>*Tick one best response*	0 Never<br>1 In the past week<br>2 In the past month<br>3 In the past 6 months<br>4 6 months ago or longer
{NMW7}	WHEN WAS THE LAST TIME THAT YOU SAW A WOMAN WITH INCOMPLETE OR UNSAFE ABORTION?<br>*Tick one best response*	0 Never<br>1 In the past week<br>2 In the past month<br>3 In the past 6 months<br>4 6 months ago or longer

WHAT SYMPTOMS AND DANGER SIGNS DURING PREGNANCY, DELIVERY, AND AFTER DELIVERY WOULD MAKE YOU DISCUSS WITH THE DOCTOR?

Listen carefully. Probe for multiple responses. Do not read out list. *Tick as many box(es) as apply*

{NMW8A}	previous bad obstetric history / precious CS / previous stillbirth	
{NMW8B}	hypertension / headache / oedema / fits / (pre-)eclampsia	
{NMW8C}	anaemia / pallor / fatigue / dypnoea	
{NMW8D}	fetal distress / no fetal movement / baby does not move	
{NMW8E}	abnormal lie / position of fetus	
{NMW8F}	sepsis / bad smelling discharge / postpartum abdominal pain	
{NMW8G}	slight bleeding / spotting	

{NMW8H}	haemorrhage / heavy bleeding	
{NMW8J}	twins / large abdomen	
{NMW8K}	obstructed / prolonged labour / indication for vacuum	
{NMW8L}	asthma / fever / malaria / MV-patient	
{NMW8M}	(grand)multiparity / premature labour / abnormal baby	
{NMW8N}	other - specify:	

{NMW9A}	ABOUT HOW MANY PATIENTS WITH INCOMPLETE OR UNSAFE ABORTION DID YOU SEE LAST MONTH? *Enter number; if none enter 0*	
{NMW9B}	HOW OLD WAS YOUR LAST PATIENT WITH INCOMPLETE OR UNSAFE ABORTION? *Enter age in years* *if not known enter 0*	
{NMW9C}	DO YOU ALWAYS GIVE INFORMATION ABOUT FAMILY PLANNING TO PATIENTS WITH INCOMPLETE OR UNSAFE ABORTION? *Tick one box*	1 Yes 0 No
{NMW9D}	IN SMRU, ABOUT HOW MANY DEATHS FROM INCOMPLETE OR UNSAFE ABORTION HAPPEN EACH YEAR? *Enter number; if none enter 0*	
{NMW9E}	DO YOU THINK THAT INCOMPLETE OR UNSAFE ABORTION IS A PROBLEM IN MAE LA CAMP (FOR THE PATIENT AND HER FAMILY)? *Tick one box*	1 Yes 0 No
{NMW10A}	DO PATIENTS YOUNGER THAN 18 YEARS NEED PERMISSION FROM PARENTS OR HUSBAND FOR ANTENATAL, DELIVERY OR POSTPARTUM SERVICES? *Tick one box*	1 Yes 0 No
{NMW10B}	DO YOU KNOW FAMILLY PLANNING SERVICES TO WHICH YOU CAN REFER PATIENTS? *Tick one box*	1 Yes 0 No
{NMW11A}	WHAT IS YOUR HIGHEST LEVEL OF EDUCATION (BEFORE SMRU)? *Tick one box*	0 No education<br>1 Primary school<br>2 Middle school<br>3 High school<br>4 Nurse school<br>5 Midwife school<br>6 University<br>7 Other - specify:
{NMW11B}	WHAT IS YOUR LEVEL AS MIDWIFE? *Tick one box*	1 Senior midwife<br>2 Junior midwife<br>3 Midwife assistant<br>4 Volunteer<br>5 Other - specify:
{NMW12}	WHEN WAS THE LAST TIME THAT YOU RECEIVED ANY MIDWIFE TRAINING IN SMRU? *Tick one best response* *If no training received, go to question NMW14*	0 Never<br>1 In the past week<br>2 In the past month<br>3 In the past 6 months<br>4 In the past year<br>9 Do not know
{NMW13}	WHEN YOU RECEIVED THIS MIDWIFE TRAINING, DID IT INCLUDE "HANDS-ON" PRACTICAL TRAINING? *Tick one best response*	1 Yes<br>0 No
{NMW14}	WHEN WAS THE LAST TIME THAT YOU RECEIVED TRAINING IN FAMILY PLANNING? *Tick one best response*	0 Never<br>1 In the past week<br>2 In the past month<br>3 In the past 6 months<br>4 In the past year<br>9 Do not know

{NMW15}	WHEN WAS THE LAST TIME THAT YOUR SUPERVISOR PROVIDED YOU WITH PERSONAL FEEDBACK ON THE JOB?<br>*Tick one best response*	0 Never<br>1 In the past week<br>2 In the past month<br>3 In the past 6 months<br>4 In the past year<br>8 I have no supervisor<br>9 Do not know
NMW16}	IN YOUR OPINION, ARE THERE ALWAYS ENOUGH MIDWIVES ON DUTY FOR THE AMOUNT OF WORK?<br>*Tick one box*	1 Yes 0 No
NMW17}	IN YOUR OPINION, DO YOU WORK TOO LONG HOURS AT NIGHT?<br>*Tick one box*	1 Yes 0 No
NMW18}	IN YOUR OPINION, DO YOU HAVE ENOUGH DAYS OFF?<br>*Tick one box*	1 Yes 0 No
NMW19}	DO YOU FEEL SAFE / CONFIDENT DOING YOUR WORK?<br>*Tick one box*	1 Yes 0 No

THANK YOU YOUR TIME. DO YOU HAVE ANY QUESTIONS THAT YOU WOULD LIKE TO ASK?
